# Now or later: Health impacts of delaying single‐dose HPV vaccine implementation in a high‐burden setting

**DOI:** 10.1002/ijc.34054

**Published:** 2022-05-24

**Authors:** Emily A. Burger, Jean‐François Laprise, Stephen Sy, Mary Caroline Regan, Kiesha Prem, Mark Jit, Marc Brisson, Jane J. Kim

**Affiliations:** ^1^ Center for Health Decision Science Harvard University T.H. Chan School of Public Health Boston Massachusetts USA; ^2^ Health Management and Health Economics University of Oslo Faculty of Medicine Oslo Norway; ^3^ Centre de Recherche du CHU de Québec Université Laval Quebec Canada; ^4^ Department of Infectious Disease Epidemiology, Faculty of Epidemiology and Population Health London School of Hygiene & Tropical Medicine London UK; ^5^ Saw Swee Hock School of Public Health National University of Singapore and National University Health System Singapore Singapore; ^6^ School of Public Health University of Hong Kong Hong Kong Special Administrative Region China; ^7^ Département de Médecine sociale et préventive, Faculté de médecine Université Laval Quebec Canada

**Keywords:** cervical cancer, human papillomavirus, simulation modeling, vaccination

## Abstract

We aimed to quantify the health impact of immediate introduction of a single‐dose human papillomavirus (HPV) vaccination program in a high‐burden setting, as waiting until forthcoming trials are completed to implement single‐dose HPV vaccination may result in health losses, particularly for cohorts who would age‐out of vaccination eligibility. Two mathematical models fitted to a high‐burden setting projected cervical cancer incidence rates associated with (a) immediate implementation of one‐dose HPV vaccination vs (b) waiting 5 years for evidence from randomized trials to determine if one‐ or two‐doses should be implemented. We conducted analyses assuming a single dose was either noninferior or inferior to two doses. The models projected that immediate implementation of a noninferior single‐dose vaccine led to a 7.2% to 9.6% increase in cancers averted between 2021 to 2120, compared to waiting 5 years. Health benefits remained greater with immediate implementation despite an inferior single‐dose efficacy (80%), but revaccination of one‐dose recipients became more important assuming vaccine waning. Under most circumstances, immediate vaccination avoided health losses for those aging out of vaccine eligibility, leading to greater health benefits than waiting for more information in 5 years.

## What's new?

Vaccination against human papillomavirus (HPV) is highly effective in preventing cervical cancer. Nonetheless, low‐income countries with high cervical cancer burden suffer from low HPV vaccination rates. Here, models were developed to quantify health impacts associated with immediate implementation of single‐dose HPV vaccination in high‐burden settings, versus waiting 5 years, when trials testing the efficacy of single‐dose vaccination will be completed. Modeling demonstrated that immediate use of single‐dose vaccine increased the number of averted cervical cancers from 2021 to 2120, compared to waiting 5 years. Thus, immediate vaccination can benefit the health of individuals soon aging out of vaccine eligibility.

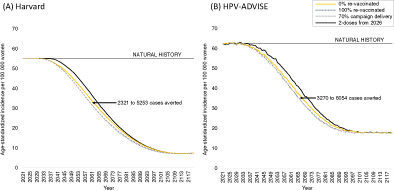

**Abbreviations**
HPV: human papillomavirus
MAC: multiage cohort
RCT: randomized controlled trial

## 1 INTRODUCTION

Prophylactic human papillomavirus (HPV) vaccination prevents cervical cancer,^1^ and is necessary to achieving the World Health Organization's (WHO) goal of eliminating cervical cancer as a public health problem.^2^ However, HPV vaccination has not been introduced in many low‐income countries with the highest burden of cervical cancer, which may be partially due to financial and logistical barriers to obtaining and delivering the preadolescent two‐dose vaccination schedule.^3^ Although guidelines for HPV vaccination recommend two doses for girls aged 9 to 14 years old,^4^ evidence from six nested observational studies have demonstrated that a single‐dose regimen may provide similar protection as two doses against HPV infection and its sequelae.^5^ Importantly, single‐dose efficacy is unlikely to be less than 85%. However, evidence of a noninferior efficacy of a single‐dose schedule in large prospectively designed randomized controlled trials (RCTs), for example, the Costa Rica ESCUDDO trial,^6^ is not expected until ~2025/2026.^5^ Despite promising signs of a robust immune response, single‐dose efficacy is a key determinant of long‐term health impact and cost‐effectiveness of alternative dosing schedules.^7^, ^8^ Before implementing a single‐dose HPV vaccination program, countries must decide whether to wait for the completion of prospective single‐dose trials. However, waiting 5 years until the trials are completed (~2026) to implement HPV vaccination may result in health losses, particularly for cohorts who would age‐out of vaccination eligibility, that is, older than age 14 years, by the time the trial results would be available.

Given the decades‐long natural history of HPV infection to cervical cancer, understanding the timing of the future cervical cancer burden under alternative implementation scenarios requires the use of mathematical simulation models, which have been used to support the planning of the WHO's elimination goals.^2^ Using a comparative model‐based approach in a high‐burden setting without ongoing HPV vaccination, we quantified the health impacts of introducing a single‐dose HPV vaccination program prior to confirmatory RCTs (eg, ESCUDDO trial results) compared to delaying HPV vaccine implementation until trial results are available, under different assumptions of single‐dose vaccine efficacy and duration.

## 2 METHODS

### 2.1 Analysis and scenarios

We used two independently developed simulation models (Harvard [Harvard T.H. Chan School of Public Health] and HPV‐ADVISE [Université Laval]) that involved dynamic modeling to capture vaccine‐related changes in HPV‐induced cervical cancer over time, including herd effects. The Harvard and HPV‐ADVISE models were used to project the change in health outcomes associated with an immediate vs a 5‐year delayed adoption of single‐dose HPV vaccination in a high‐burden setting (age‐standardized rate >50 per 100 000 women) without an ongoing HPV vaccination program under alternative single‐dose efficacy and duration profiles.

To reflect the uncertainty of forthcoming trial results, we conducted two separate analyses assuming that efficacy associated with a single dose was either noninferior or inferior to two doses (Table S1). For “Analysis 1,” we compared a noninferior single‐dose vaccine implemented in year 2021 with delayed implementation of a noninferior (100% efficacy) single‐dose vaccine in 2026 (after the trial results presumably established the noninferior efficacy). Analysis 1 essentially quantifies the health impacts of a 5‐year delayed implementation of a noninferior single‐dose HPV vaccine, which assumes a loss of direct vaccine protection for the girls aged 10 to 14 in 2021 (as they age out of vaccine eligibility by 2026) and to a lesser extent for those girls turning age nine between 2021 and 2025 who would be relatively older at the time of their “delayed” vaccination in 2026 (Figure S1). For “Analysis 2,” we assumed the single‐dose vaccine is ultimately found to be inferior (80% efficacy) in 2026 but implementation of a single‐dose regimen had moved forward in 2021, resulting in routine programs reverting to a two‐dose schedule in 2026 (after the timepoint at which the trial results presumably established the inferior efficacy). For girls previously vaccinated with an inferior vaccine, we explored alternative revaccination scenarios that varied by the coverage of the second dose and target age group (Table S1 and Figure S2).

### 2.2 Simulation models

The Harvard and HPV‐ADVISE models, which have been described in detail previously,^2^ capture HPV natural history and cervical disease, as well as HPV transmission. Both models underwent calibration to reflect sexual behavior, HPV prevalence and cervical cancer burden from a high‐burden setting (age‐standardized incidence rate of >50 per 100 000 women), such as Uganda. These models are particularly suited to capture the important dynamics in this analysis as they (a) track intercohort effects to project whether a vaccine with <100% efficacy can be compensated for through herd effects, and (b) they calibrated the age‐specific force of infection to local cancer epidemiology to explore whether a vaccine that wanes will protect for long enough to avoid most cancers.

As previously described,^2^, ^7^ the Harvard model uses a multimodel approach to project the population health consequences of alternative cervical cancer scenarios over time. For the current analysis, the multimodeling approach involved the dynamic model of HPV transmission (“Harvard‐HPV”), and the stochastic model of cervical carcinogenesis (“Harvard‐CC”) from the highest‐burden epidemiological profile used in the Harvard model WHO elimination analysis.^2^ Harvard‐HPV is an individual (ie, agent‐based) model that includes seven independent HPV genotypes (HPV‐16, ‐18, ‐31, ‐33, ‐45, ‐52 and ‐58). The model projects reductions in HPV incidence by genotype over time associated with each cancer control strategy; these reductions served as inputs into Harvard‐CC. Harvard‐CC is an individual‐based model that tracks women from age 9 years as they transition through cervical cancer‐related health states until death.^9^ Harvard‐CC was used to project cervical cancer incidence by age over time for each scenario.

HPV‐ADVISE LMIC^2^, ^8^ is an individual‐based, transmission‐dynamic model of multitype HPV infection (18 HPV types modeled separately, including vaccine types 6/11/16/18/31/33/45/52/58) and related diseases (see Technical Appendix: http://www.marc-brisson.net/HPVadvise-LMIC.pdf). The model reproduces demographic characteristics, sexual behavior and transmission of HPV, natural history of HPV‐associated diseases (HPV infection, natural immunity, three grades of cervical lesions and three cervical cancer stages), screening and treatment. Transmission is gender‐ and age‐specific, and depends on sexual behavior (eg, mixing patterns), HPV biology and natural history (eg, probability of transmission and natural immunity). HPV‐ADVISE was used to generate the mean age‐specific incidence of cervical cancer over time for each vaccination scenario across the 50 good‐fitting natural history parameter sets.

### 2.3 Assumptions and outcomes

For both analyses, we assumed immediate implementation involved a single‐dose regimen starting in 2021, and delayed implementation involved whichever high‐efficacy regimen from 2026 onward—that is, either continuing with the noninferior single‐dose schedule or switching to a two‐dose schedule—depending on the outcome of the clinical trials. All scenarios also assumed that the first year of the vaccination program (either 2021 or 2026) involved routine (ongoing) vaccination for girls aged 9 years and a 1‐year multiage cohort (MAC) vaccination for girls aged 10 to 14 years. For the noninferior single‐dose analysis (Analysis 1), we assumed 100% lifelong protection against seven high‐risk HPV (hrHPV) types (HPV‐16, ‐18, ‐31, ‐33, ‐45, ‐52 and ‐58), while an inferior single‐dose vaccine (Analysis 2) achieved 80% lifelong vaccine efficacy (based on the lower‐bound target efficacy for one‐dose HPV vaccination in ESCUUDO^6^). In sensitivity analysis, we explored an inferior vaccine with 80% efficacy that waned after 20 years (normally distributed with a SD of 5 years). Although each model incorporated vaccine efficacy differently (vaccine “degree” for Harvard and vaccine “take” for HPV‐ADVISE), both models achieved an 80% cumulative reduction in vaccine‐type HPV infections for a vaccinated cohort at year five (vaccine degree parameter calibrated in the Harvard model). The two‐dose schedule was assumed to achieve 100% lifelong protection for the same seven hrHPV types. We assumed the vaccine protected against incident infections and would not affect clearance of a prevalent infection already present at the time of vaccine receipt.

For vaccination coverage, we assumed that the first dose reached 70% of girls aged 9 years, and 70% of 10‐ to 14‐year‐olds in the first year of the vaccination program. In the case of an inferior one‐dose vaccine regimen (Analysis 2), we explored several revaccination scenarios: (a) an optimistic revaccination scenario assuming the entire proportion of the 10 cohorts of girls that were previously given a single inferior dose were identified and given a second dose to boost protection, that is, 100% revaccination coverage; (b) a pessimistic scenario which assumed it was programmatically impossible to find and revaccinate anyone who had been vaccinated prior to 2026 and (c) a pragmatic scenario which assumed a MAC (ages 10‐14) “revaccination” campaign delivery approach (Figure S2c). For this scenario we assumed a 70% random coverage that reached 49% of all girls aged 10 to 14 in 2026 who had previously received only a single dose, as well as 21% of unvaccinated girls. As vaccinating girls aged >14 years may be logistically difficult to revaccinate than girls aged ≤14 years in some settings,^10^ the “second opportunity” to be vaccinated in this scenario was not performed in a targeted manner and only to those that would have been age‐eligible for the 10‐ to 14‐year‐old MAC under a 2026 “delayed” implementation. In total, under the two waning assumptions (lifelong and 20 years), we evaluated six inferior single‐dose vaccination scenarios in Analysis 2 (Table S1; Figure S2).

Analysis outcomes included age‐standardized (WHO 2015 female population^11^) cervical cancer incidence rates per 100 000 women and the number of cervical cancer cases between 2021 to 2120 (inclusive). For cases, we assumed a starting population of 1 million women alive in 2021 with population growth (average 1.6% per year) and age distribution similar to projections for Uganda^11^ (Table S2). Cases were quantified for all cohorts alive over the analytic period to capture direct and indirect benefits, but also for the five birth cohorts that would age‐out under a delayed 2026 implementation, that is, those girls aged 10 to 14 in 2021 (results for the Harvard model only).

### 2.4 Role of funding source

The funders had no role in the study design; in the collection, analysis and interpretation of data; in the writing of the report; and in the decision to submit the article for publication.

## 3 RESULTS

### 3.1 Noninferior single‐dose HPV vaccine (Analysis 1)

**FIGURE 1:**
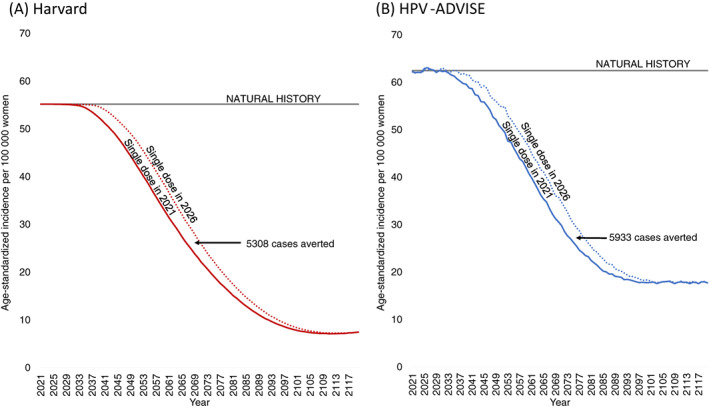
Age‐standardized incidence per 100 000 women over time for a noninferior single dose human papillomavirus (HPV) vaccination program implemented in 2021 or delayed to 2026 for the Harvard (A) and HPV‐ADVISE (B) simulation models. Cases averted are compared to a noninferior single‐dose vaccination program implemented in 2026 and are estimated for a starting cohort of 1 million women alive in 2021 over years 2021 to 2120 (inclusive). [Color figure can be viewed at wileyonlinelibrary.com]

When we assumed a single‐dose vaccine regimen was noninferior compared to a two‐dose regimen (100% lifelong efficacy), the two models projected that immediate implementation of single‐dose vaccination was expected to expedite reductions in age‐standardized cervical cancer rates and would avert between 5308 and 5933 additional cases (7.2%‐9.6% increase) over 2021 to 2120 for a starting population of 1 million women alive in 2021, compared to delayed HPV vaccine implementation in five years (Figure 1; Tables S3 and S4). Over 50% of these averted cases were concentrated among the 10‐ to 14‐year‐old 2021 cohort who would have aged‐out of vaccine eligibility under a delayed 2026 vaccination policy (Figure S3). Importantly, the more immediate introduction of HPV vaccination with a noninferior single dose would not change the final equilibrium rate in either model after 80 years (7.5 per 100 000 women for Harvard; 18.0 per 100 000 women for HPV‐ADVISE), reflecting an overall 72% to 86% reduction in cancer incidence compared to natural history.

### 3.2 Inferior single‐dose HPV vaccine (Analysis 2)

**FIGURE 2:**
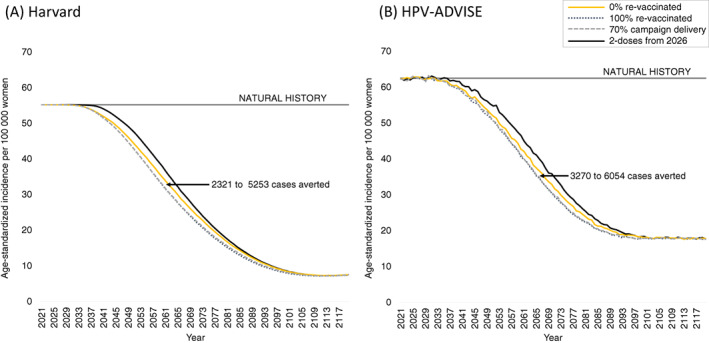
Age‐standardized incidence per 100 000 women over time by revaccination approach for an inferior (80% lifelong efficacy) single‐dose human papillomavirus (HPV) vaccination program implemented in 2021 with a switch to a two‐dose program in 2026 compared to a delayed two‐dose program implemented in 2026 (see Table S1 and Figure S2 for scenarios) for the Harvard (A) and HPV‐ADVISE (B) simulation models. Cases averted are compared to a two‐dose vaccination program implemented in 2026 and are estimated for a starting cohort of 1 million women alive in 2021 over years 2021 to 2120 (inclusive) (see Table S2). Stochastic noise in HPV‐ADVISE leads to a small increase in the number of cases averted for the 100% revaccination scenario compared to the cases averted shown in Figure 1. [Color figure can be viewed at wileyonlinelibrary.com]

When we assumed a single‐dose vaccine regimen is inferior to two doses (80% lifelong efficacy), prompting a switch to a two‐dose program five years after introduction of a single dose, we found that starting a single‐dose vaccine program immediately yielded positive health benefits compared to waiting to implement a two‐dose program in 2026 (Figure 2); the range of benefits varied by revaccination scenario. For example, across the two models, immediate implementation of single‐dose HPV vaccination averted between 2321 and 3270 additional cases (4.0%‐4.2% increase) with 0% revaccination and 5253 to 6000 additional cases (7.3%‐9.5% increase) with 100% revaccination over 2021 to 2120 for a starting population of 1 million women alive in 2021 compared to delayed implementation. In sensitivity analysis when we assumed a single‐dose vaccine was both less efficacious (80%) and provided only 20 years of protection, the HPV‐ADVISE model continued to find positive health benefits for all revaccination scenarios compared to delayed implementation (Figure S4). In contrast, for the Harvard model, a 2026 revaccination scenario after a 2021 single‐dose implementation would need to achieve at least 50% coverage of the 10‐ to 19‐year‐olds in 2026 to provide positive health benefits compared to a delayed two‐dose program (break‐even analysis results not shown).

## 4 DISCUSSION

In this comparative health impact modeling analysis, we found that early implementation of a single‐dose HPV vaccination schedule ahead of confirmatory trials was likely to yield greater health benefits than waiting for the completion of trial results prior to implementation. Even under scenarios of an inferior 80% (lifelong) single‐dose efficacy, which is unlikely given current evidence,^5^ we found that nearly all benefits of early implementation would be maintained across a range of revaccination scenarios. Importantly, even if a program was unable to revaccinate any of the girls previously given a single dose, we found that there were still positive health benefits of starting vaccination early. These health benefits primarily stem from providing the 10‐ to 14‐year‐olds in 2021 an opportunity to be vaccinated (Figure S3).

Our findings of the potential health losses associated with delaying HPV vaccination implementation are set within the context of a global COVID‐19 pandemic, which may further delay the completion of key randomized trials, disrupt the implementation of HPV vaccination programs, or further limit vaccine supply.^12^ Our findings of the potential losses due to delays can generally be representative of a 5‐year delay in implementing a population‐based vaccination program when there are upper age‐eligibility thresholds for vaccine receipt, that is, age 14 years. Importantly, our findings support previous findings that limited supplies of the HPV vaccine should be prioritized to girls that age‐out of eligibility under implementation delays, for example, through implementation of a “reverse MAC” (ie, delaying the age of routine vaccination to 14 years (with or without a subsequent switch to routine vaccination at age 9 years), which has been shown to maximize health benefits of a limited supply of HPV vaccine doses.^8^ Furthermore, assuming an inferior but lifelong single‐dose HPV vaccine, a revaccination MAC campaign approach averted nearly as many cancer cases as revaccinating 100% of previously vaccinated women, which is likely due to the health benefits associated with providing a second opportunity for unvaccinated women to receive their first HPV vaccine dose (effectively increasing vaccine coverage).

Several limitations are worth noting. We did not consider the health benefits beyond cervical cancer; therefore, we have likely underestimated the potential health benefits of a timely implementation of HPV vaccination program in terms of wider prevention of HPV‐related cancers. Although we considered a scenario of population growth associated with a low‐income country, projections over the next century face inherent limitations. To identify the greatest possible impact of 70% coverage with a two‐dose vaccination schedule, we assumed there was no loss to follow‐up between the first and second dose; however, in practice, 100% completion of both doses is unlikely. Therefore, the proportion of women who do not complete the two‐dose series (ie, received a single dose) would reduce the overall effectiveness of our two‐dose comparator in Analysis 2. In addition, we did not consider the impact of a potential scale‐up or ongoing cervical cancer screening program, which could blunt some of the health losses associated with delayed HPV vaccine implementation. Our analysis is strengthened by a comparative modeling approach using two models that have been used for a wide range of policy analyses, including the WHO cervical cancer elimination projections.^2^, ^13^ In most instances, the two models' projections are quite similar across the wide‐range of single‐dose efficacy profiles; however, there are some differences when we examined a single‐dose vaccine that is both inferior and wanes, with the Harvard model finding that revaccinating girls is important to preserve the positive health benefit of single‐dose vaccination. The differences between the Harvard and HPV‐ADVISE models are likely due to differences in assumptions of sexual behavior and HPV exposure in women past age 30 years (around when the vaccine wanes). Given current evidence, waning of a single‐dose HPV vaccine is unlikely as antibody levels have been shown to not qualitatively decline by the number of doses given between years four and 11 in the Costa Rica Vaccine Trial.^14^ Nonetheless, duration of protection will remain a key uncertainty even when forthcoming RCTs conclude in five years.

## 5 CONCLUSIONS

Under most scenarios examined, immediate implementation of a single‐dose HPV vaccination leads to greater health benefits than waiting until more information on vaccine efficacy is available from ongoing clinical trials, expected in five years. Health benefits are maximized by expediting vaccination of cohorts that would otherwise age‐out of HPV vaccine eligibility in those five years.

## AUTHOR CONTRIBUTIONS

Emily A Burger and Jane J. Kim designed the study and led overall data interpretation. Emily A Burger and Jane J. Kim led the Harvard analysis, Jean‐François Laprise and Marc Brisson led the HPV‐ADVISE analysis, and Emily A Burger and Jane J. Kim led the combined analysis. Emily A Burger, Stephen Sy, Mary Caroline Regan, Jane J. Kim, Jean‐François Laprise, Marc Brisson participated in data analyses and Emily A Burger, Stephen Sy, Mary Caroline Regan, Jane J. Kim, Jean‐François Laprise, Marc Brisson, Kiesha Prem, Mark Jit contributed to interpretation. Emily A Burger produced the figures and tables. Marc Brisson, Jane J. Kim, Emily A Burger, Mark Jit, Kiesha Prem and Jean‐François Laprise consulted on the analyses. All authors interpreted the results and critically revised the article for scientific content. All authors approved the final version of the article. The work reported in the article has been performed by the authors, unless clearly specified in the text.

## CONFLICT OF INTEREST

The authors declare no conflicts of interest.

## ETHICS STATEMENT

Ethical approval was not required because the work was carried out on published documents, aggregated data and anonymous datasets.

## Supporting information


**Appendix S1** Supporting Information.

## Data Availability

Supporting Information contained in the supplementary material of Brisson et al^2^ provides details on model inputs, calibration to epidemiologic data and calibration approach in line with good modeling practice. The data that support the findings of our study are available from the corresponding author upon reasonable request.
