# Supplementary material for: Now or later: Health impacts of delaying single‐dose HPV vaccine implementation in a high‐burden setting
Source: Int J Cancer. 2022 May 24;151(10):1804–9. doi: 10.1002/ijc.34054 (PMC9790243; doi:10.1002/ijc.34054)

Supplementary Appendix for:

Now or later: Health impacts of delaying single-dose HPV vaccine implementation in a high-burden setting

Authors:

Emily A Burger
Jean-François Laprise
Stephen Sy
Mary Caroline Regan
Kiesha Prem
Mark Jit
Marc Brisson
Jane J Kim

Table of contents:

1. Supplementary material & methods, pages 2-5
2. Supplementary results, page 6-8

1. Supplementary material & methods

Appendix Table S1. Scenarios for 'Analysis 1' (non-inferior single-dose HPV vaccine) and 'Analysis 2' (inferior single-dose HPV vaccine)

	**Scenario**	**Dosage**	**Sex**	**First dose Coverage**	**Second dose coverage for revaccination for girls aged 10-14 (in 2026)**	**Second dose coverage for revaccination for girls aged 15-19 (in 2026)**	**Efficacy**	**Vaccine duration**	**Vaccination target ages year 1**	**Vaccination Start Year**	**Vaccination policy switch year****	**Revaccination delivery approach**
Natural history	0	0	-	0			-	-	-	-		-
Analysis I: Non-inferior 1D	1a	1	F	70%	-	-	100%	Lifelong	9, 10-14	2021	-	-
	1b	1	F	70%	-	-	100%	Lifelong	9, 10-14	2026	-	-
Analysis II: Inferior 1D	2	2	F	70%	-	-	100%	Lifelong	9, 10-14	2026	-	-
	2a	1	F	70%	100%	100%	80%	Lifelong	9, 10-14	2021	2026	Targeted
	2b	1	F	70%	0%	0%	80%	Lifelong	9, 10-14	2021	2026	--
	2c	1	F	70%	70%*	0%	80%	Lifelong	9, 10-14	2021	2026	Campaign
	2d	1	F	70%	100%	100%	80%	20	9, 10-14	2021	2026	Targeted
	2e	1	F	70%	0%	0%	80%	20	9, 10-14	2021	2026	--
	2f	1	F	70%	70%*	0%	80%	20	9, 10-14	2021	2026	Campaign

*70% coverage applied to both previously vaccinated and unvaccinated girls; **For routine program and previously vaccinated girls, if applicable

Appendix Figure S1. Single-dose implementation in 2021 with a non-inferior vaccine (A) and 'delayed' single-dose implementation in 2026 (B).

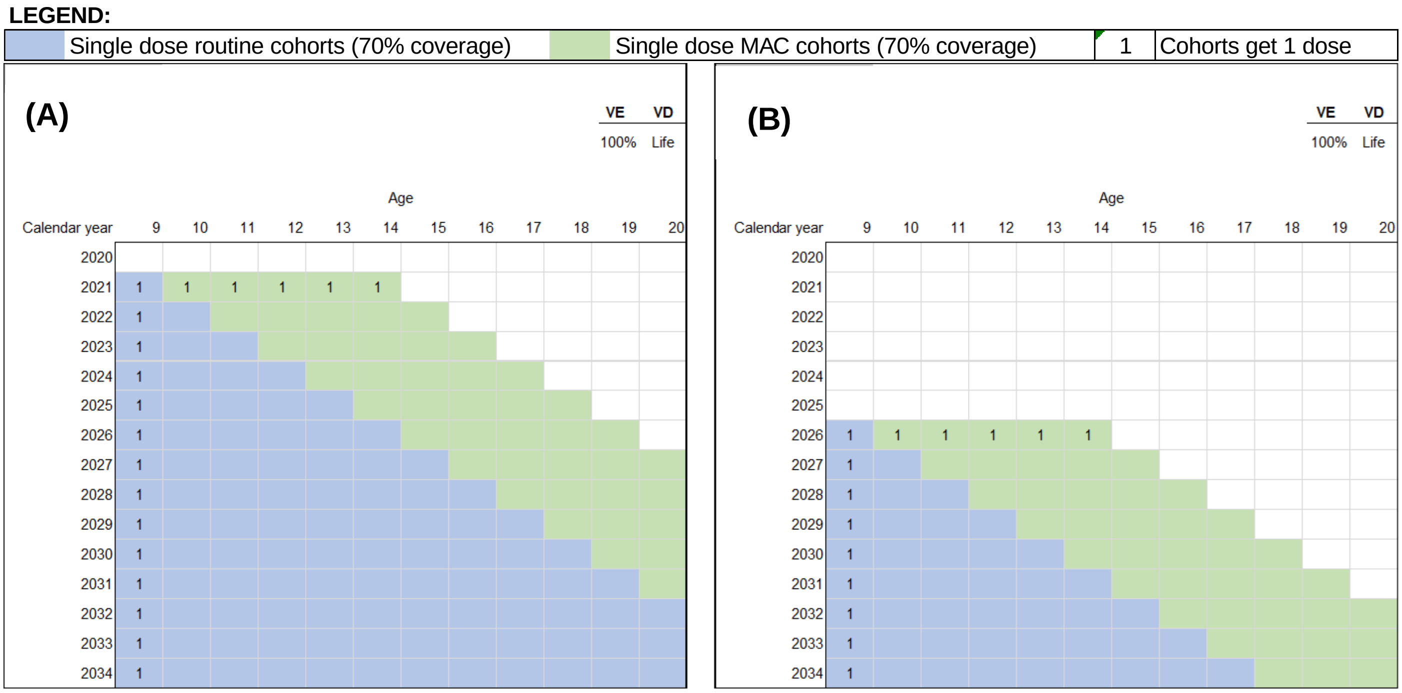

Appendix Figure S2. Single-dose implementation in 2021 with an inferior vaccine assuming 100% re-vaccination in 2026 (A), 0% revaccination in 2026 (B), campaign delivery revaccination in 2026 (C) and 2-dose delayed vaccination in 2026 (D).

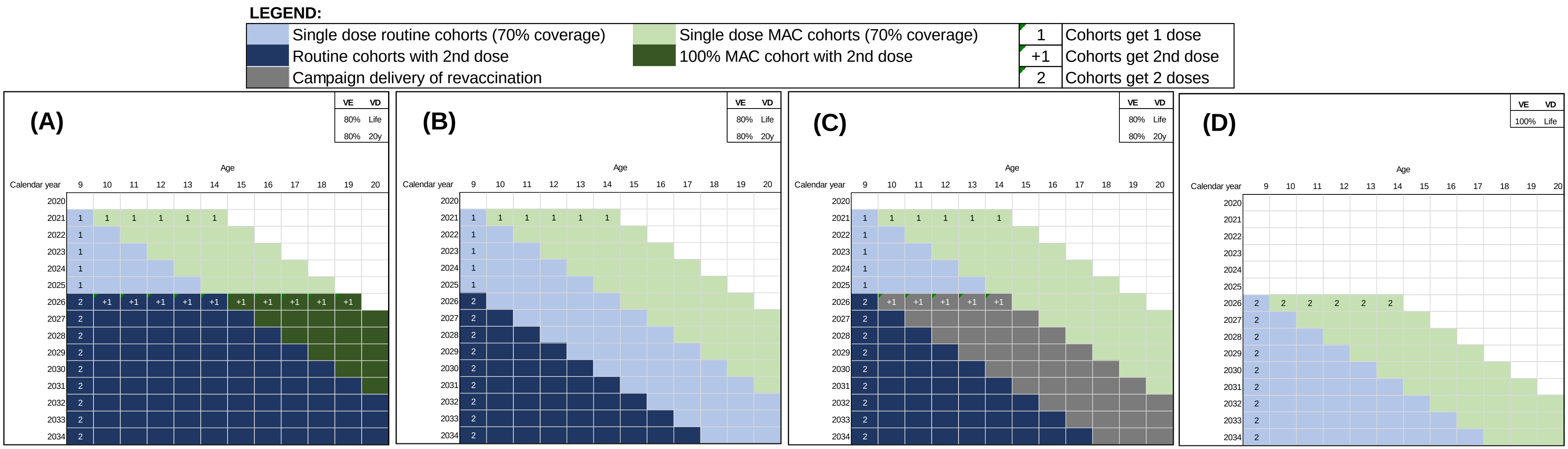

Appendix Table S2. Standardized 1 million population in 2021 with growth (to 2120) used to estimate cases averted (see methods)

	Year		
**Age**	**2021**	**…**	**2120**
0	37046.04	…	58027.94
1	36073.72	…	58492.39
2	35008.35	…	58923.59
3	33991.76	…	59321.71
4	33019.85	…	59222.75
5	32088.49	…	59647.95
6	31193.15	…	60050.73
7	30329.38	…	60433.2
8	29494.91	…	60795.89
9	28686.4	…	61140.99
10	27898.58	…	61469.13
11	27129.68	…	61780.96
12	26380.21	…	62078.1
13	25622.92	…	62363.74
14	24844.01	…	62620.94
15	24052.92	…	62848.72
16	23277.28	…	63167.45
17	22517.05	…	63520.03
18	21745.67	…	63806.56
19	20954.49	…	63978.72
20	20156.86	…	64156.02
21	19372.1	…	64293.31
22	18593.23	…	64402.49
23	17856.23	…	64527.31
24	17179.97	…	64679.75
25	16547.41	…	64804.78
26	15925.2	…	64954.02
27	15322.9	…	65115.32
28	14719.78	…	65248.48
29	14103.12	…	65335.04
30	13485.41	…	65419.6
31	12889.93	…	65448.12
32	12309.71	…	65444.13
33	11763.98	…	65456.33
34	11264.01	…	65516.14
35	10799.02	…	65560.78
36	10345.94	…	65556.78
37	9908.016	…	65518
38	9483.341	…	65467.09
39	9068.152	…	65412.59
40	8663.06	…	65332.82
41	8272.971	…	65307.35
42	7897.395	…	65316.44
43	7526.602	…	65289.57
44	7156.423	…	65164.14
45	6790.331	…	65012.2
46	6437.158	…	64928.73
47	6096.209	…	64839.15
48	5763.845	…	64659.07
49	5439.452	…	64379.93
50	5125.075	…	64107.37
51	4823.29	…	63655.17
52	4533.647	…	63113.86
53	4261.214	…	62645.42
54	4008.772	…	62358.01
55	3774.523	…	62037.76
56	3552.047	…	61534.34
57	3340.283	…	60984.02
58	3149.817	…	60525.25
59	2984.493	…	60235.57
60	2838.629	…	59930.73
61	2701.023	…	59468.75
62	2571.879	…	58899.88
63	2450.257	…	58318.15
64	2333.95	…	57752.27
65	2221.609	…	57094.72
66	2115.685	…	56791.12
67	2017.448	…	56627.86
68	1909.44	…	56213.27
69	1783.852	…	55219.46
70	1648.249	…	54147.8
71	1517.675	…	53579.62
72	1388.572	…	53135.51
73	1268.994	…	52310.87
74	1164.338	…	50690.68
75	1070.107	…	48958.18
76	977.8381	…	47852.56
77	890.6385	…	46828.05
78	801.2312	…	45219.27
79	705.6509	…	42475.67
80	608.5989	…	39537.62
81	517.6381	…	37761.14
82	429.9888	…	36416.8
83	356.8523	…	34372.88
84	304.0746	…	31079.05
85	265.9324	…	27997.55
86	230.4066	…	23737.25
87	201.2583	…	19269.98
88	172.437	…	15854.5
89	140.0182	…	18705.49
90	106.373	…	5714.664
91	74.19943	…	4749.786
92	51.87828	…	4058.975
93	41.8215	…	3215.104
94	30.33387	…	2218.215
95	17.4154	…	1608.635
96	10.95617	…	1387.836
97	8.462414	…	1125.624
98	5.723372	…	822.0398
99	2.779924	…	477.0839
Sum	1000000	…	5179058

2. Supplementary results

Appendix Table S3. Cumulative number of cases over 2021-2120 by scenario (See Appendix Table S1) for the Harvard model

	Scenario	Cases	Averted vs S0	Averted vs S1b	% increase in averted (immediate vs delay)	Averted vs S2	% increase in averted (immediate vs delay)
**Natural history**	0	167344					
**Analysis I: Non-inferior 1D**	1a	50144	117200	5308	9.6%		
	1b	55452	111893				
**Analysis II: Inferior 1D**	2	55452	111893	--			
	2a	50199	117146			5253	9.5%
	2b	53131	114213			2321	4.2%
	2c	50730	116615			4722	8.5%
	2d	50253	117092			5199	9.4%
	2e	60417	106927			-4965	-9.0%
	2f	56565	110779			-1113	-2.0%

Appendix Table S4. Cumulative number of cases over 2021-2120 by scenario (See Appendix Table S1) for the HPV-ADVISE model

	Scenario	Cases	Averted vs S0	Averted vs S1b	% increase in averted (immediate vs delay)	Averted vs S2	% increase in averted (immediate vs delay)
**Natural history**	0	192920					
**Analysis I: Non-inferior 1D**	1a	76745	116174	5933	7.2%		
	1b	82679	110241				
**Analysis II: Inferior 1D**	2	82679	110241	--			
	2a	76624	116295			6054	7.3%
	2b	79408	113511			3270	4.0%
	2c	76988	115932			5691	6.9%
	2d	76675	116244			6003	7.3%
	2e	79277	113643			3402	4.1%
	2f	76921	115999			5758	7.0%

Appendix Figure S3. Cervical cancer cases among the 10-14 years-old in 2021 assuming a non-inferior single-dose HPV vaccination program was implemented in 2021 or delayed until 2026, i.e., 'Analysis 1' (for the Harvard model only). Cases are estimated for a starting cohort of 1 million women alive in 2021 over 2021-2120 (inclusive). For the 2026 'delay' scenario, these girls were age-ineligible for vaccination by 2026; therefore, health benefits are indirect only, i.e., herd immunity. The cohort size for the 10-14-year-old 2021 starting cohort is approximately 132,000.

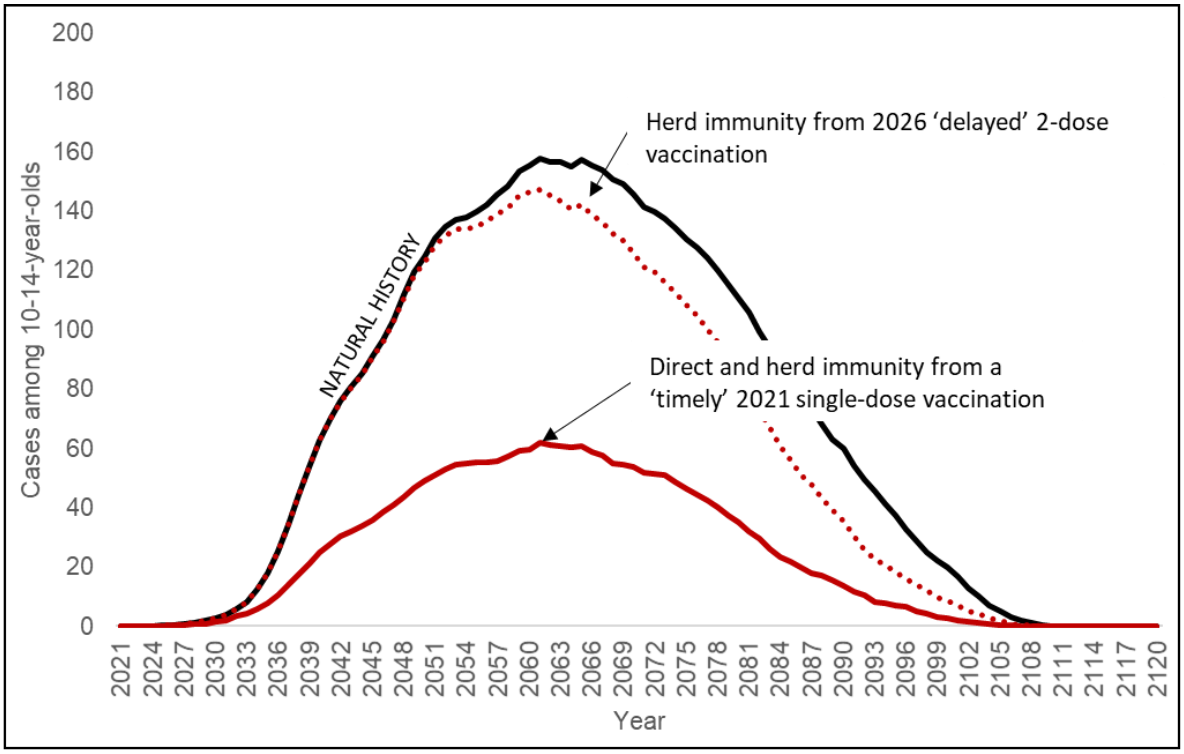

Appendix Figure S4. Age-standardized incidence per 100,000 women over time by revaccination approach for an inferior (80% efficacy with a 20-year duration (5-year standard deviation)) single dose human papillomavirus (HPV) vaccination program implemented in 2021 with a switch to a 2-dose program in 2026 compared with a delayed 2-dose program implemented in 2026 for the A) Harvard and B) HPV-ADVISE simulation models. Cases averted are compared with a 2-dose vaccination program implemented in 2026 and are estimated for a starting cohort of 1 million women alive in 2021 over 2021-2120 (inclusive). Stochastic noise in HPV-ADVISE leads to a small increase in the number of cases averted for the 100% revaccination scenario compared with the cases averted shown in main manuscript **Figure 1**.

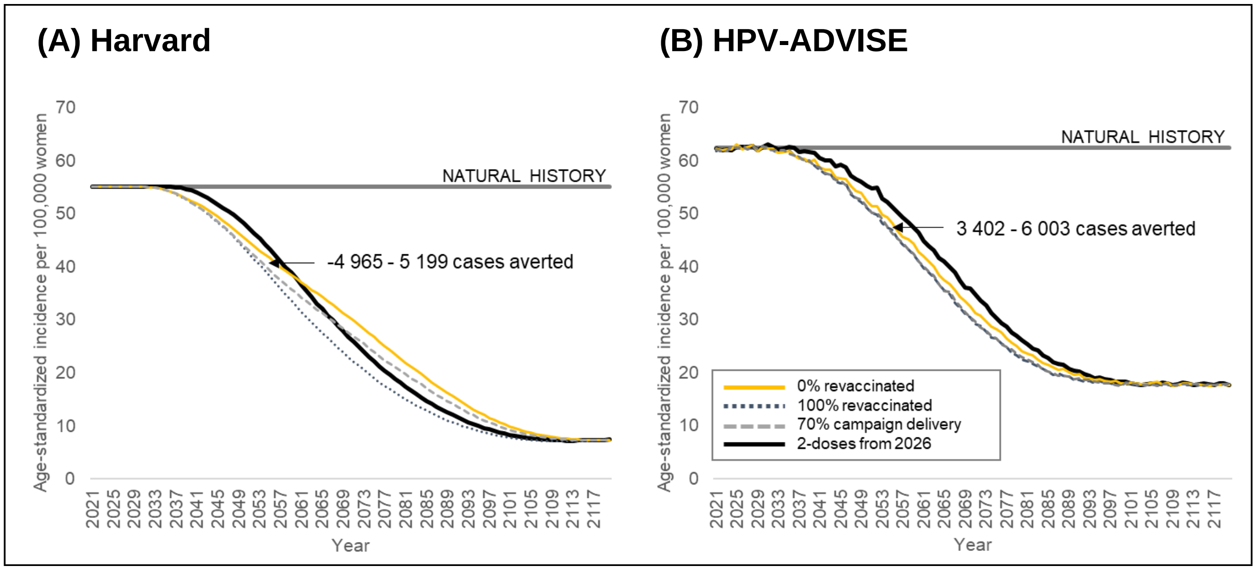

Supplement: Supplementary file 1 — Appendix S1 Supporting Information. [file IJC-151-1804-s001.pdf]
